# Social disparities in the prevalence of multimorbidity – A register-based population study

**DOI:** 10.1186/s12889-017-4314-8

**Published:** 2017-05-10

**Authors:** Michaela L. Schiøtz, Anders Stockmarr, Dorte Høst, Charlotte Glümer, Anne Frølich

**Affiliations:** 1 Intersectoral Research Unit for Health Services, The Danish Capital Region, Bispebjerg Bakke 23, Building 20D, 2nd Floor, DK, -2400 Copenhagen, NV Denmark; 2 Research Unit for Chronic Conditions, Bispebjerg University Hospital, Bispebjerg Bakke 23, Building 20D, 2nd Floor, DK, -2400 Copenhagen, NV Denmark; 3 Department of Applied Mathematics and Computer Science, Technical University of Denmark Richard Petersens Plads, Building 324, 2800 Kongens Lyngby, Denmark; 4 Research Centre for Prevention and Health, The Capital Region, Rigshospitalet, Copenhagen University, Nordre Ringvej 57 Building 84-85, 2600 Glostrup, DK Denmark; 5 Department of Health Science and Technology, Aalborg University, Frederik Bajers Vej 5, Aalborg, 9220 Denmark

**Keywords:** Multimorbidity, Epidemiology, Prevalence, Socioeconomic factors

## Abstract

**Background:**

Prevalences of multimorbidity vary between European studies and several methods and definitions are used. In this study we examine the prevalence of multimorbidity in relation to age, gender and educational attainment and the association between physical and mental health conditions and educational attainment in a Danish population.

**Methods:**

A cross-sectional design was used to study the prevalence of multimorbidity, defined as two or more chronic conditions, and of comorbid physical and mental health conditions across age groups and educational attainment levels among 1,397,173 individuals aged 16 years and older who lived in the Capital Region of Denmark on January 1st, 2012. After calculating prevalence, odds ratios for multimorbidity and mental health conditions were derived from logistic regression on gender, age, age squared, education and number of physical conditions (only for odds ratios for mental health conditions). Odds ratios for having multimorbidity and mental health conditions for each variable were adjusted for all other variables.

**Results:**

Multimorbidity prevalence was 21.6%. Half of the population aged 65 and above had multimorbidity, and prevalence was inversely related to educational attainment: 26.9% (95% CI, 26.8–26.9) among those with lower secondary education versus 13.5% (95% CI, 13.5–13.6) among people with postgraduate education. Adjusted odds ratios for multimorbidity were 0.50 (95% CI, 0.49–0.51) for people with postgraduate education, compared to people with lower secondary education. Among all population members, 4.9% (95% CI, 4.9–4.9) had both a physical and a mental health condition, a proportion that increased to 22.6% of people with multimorbidity. Physical and mental health comorbidity was more prevalent in women (6.33%; 95% CI, 6.3–6.4) than men (3.34%; 95% CI, 3.3–3.4) and approximately 50 times more prevalent among older persons than younger ones. Physical and mental health comorbidity was also twice as prevalent among people with lower secondary education than among those with postgraduate education. The presence of a mental health condition was strongly associated with the number of physical conditions; those with five or more physical conditions had an adjusted odds ratio for a mental health condition of 3.93 (95% CI, 3.8–4.1), compared to those with no physical conditions.

**Conclusion:**

Multimorbidity prevalence and patterns in the Danish population are comparable to those of other European populations. The high prevalence of mental and physical health conditions highlights the need to ensure that healthcare systems deliver care that takes physical and mental comorbidity into account. Further, the higher prevalence of multimorbidity among persons with low educational attainment emphasizes the importance of having a health care system providing care that is beneficial to all regardless of socioeconomic status.

## Background

The increasing number of people living with multiple chronic conditions, also known as multimorbidity, is a growing public health problem and a challenge for current and future healthcare systems [1]. In addition, multimorbidity is associated with increased risk of mortality, reduced functional status and increased use of healthcare services [2]. Recent studies show that the prevalence of multimorbidity is increasing, rises with age [3] and is higher in socioeconomically deprived groups [4]. However, there are marked variations among studies of the prevalence of multimorbidity with respect to both methodology and findings [3]. Numerous definitions of multimorbidity have been used [5]. According to Almirall et al., the most frequent definition of multimorbidity was “more than one or multiple chronic or long-term diseases/conditions” (including physical or mental diseases or both), which was closely followed by “more than one disease or condition” without specifying chronic or long-term duration [5]. The former is consistent with the definition used by the World Health Organization, which defines multimorbidity as “being affected by two or more chronic health conditions” [6]. Furthermore, methods used to identify persons with multimorbidity vary across prevalence studies. A key issue is how many conditions should be studied [7]. Recent reviews conclude that a minimum of 11[8] or 12 [3] conditions should be included when measuring multimorbidity prevalence.

The prevalence of multimorbidity, defined as two or more chronic conditions, identified in European studies varies substantially from 72.7% of a Portuguese population with two or more of 147 listed chronic conditions [9] to 13% of a Dutch population with two or more of 29 listed conditions [10]. These large differences in the prevalence of multimorbidity may to some extent be explained by the use of different methodological approaches both with regard to numbers of conditions included as well as whether self-reported questionnaires, data from patient records or registers are used. Also, the differences in prevalence of multimorbidity may reflect real differences among different European populations. In Denmark, central registries including information from the health care system as well as information about socioeconomic status make it possible to obtain data from an entire population. Information about the prevalence of chronic conditions based on validated algorithms can be obtained from the registries [11]. Thus, it is possible to study prevalence and patterns of multimorbidity related to socioeconomic status in a full population. In a recent study focusing on the prevalence of multimorbidity in relation to age and socioeconomic deprivation in a Scottish population, Barnett et al. found that 23.2% of all patients had two or more of 40 selected conditions and that the onset of multimorbidity occurred 10–15 years earlier in people living in the most socioeconomically deprived areas; deprivation was particularly associated with multimorbidity that included mental health conditions [4]. Additionally, it was found that some conditions were more prevalent as comorbidities among people living in deprived areas [4]. Likewise, other studies find that mental health conditions often coexist with chronic physical health conditions [4, 12–14], leading to multimorbidity that includes mental health conditions. Multimorbidity that includes mental health conditions has been associated with greater symptom burden and functional impairment, poorer quality of life, higher costs and excess mortality [15, 16]. However, only a few studies have examined the prevalence and patterns of co-occurrence of mental health conditions across the entire life span using medical record data [4, 17, 18].

Thus, in order to examine how prevalence and patterns of multimorbidity in the Danish population compare to prevalence and patterns of multimorbidity in other European populations, the aim of the study was to examine: 1) the prevalence of multimorbidity in relation to age, gender and socioeconomic status; 2) the association between physical and mental health conditions and educational attainment; and 3) the prevalence of selected pairs of chronic conditions by socioeconomic status.

## Methods

Using a cross-sectional design, we studied the prevalence of multimorbidity and of comorbid physical and mental health conditions across age groups and educational attainment levels. The study population consisted of all individuals aged16 years and older who lived in the Danish Capital Region on January 1st 2012 (*N* = 1,397,173), representing one third of the entire Danish population aged 16 years and older. Information about chronic conditions, use of healthcare services and demographics, including gender, age, educational level and place of residence, were extracted from national registers and health registers. National registers do not provide direct information about chronic conditions diagnosed in the primary sector. We used diagnostic algorithms developed by the Research Center for Prevention and Health at Glostrup University Hospital to identify 16 selected chronic conditions diagnosed in both primary and secondary healthcare sectors [11]. The algorithms use data from the Danish National Patient Register [19], the Danish National Prescription Registry [20], the Danish National Health Service Register [21] and The National Diabetes Register [22] to obtain information about use of healthcare services, purchase of prescribed medications and ICD-10 codes. Chronic conditions included diabetes, cancer, chronic back pain, osteoarthritis, osteoporosis, joint disease, allergies, chronic obstructive pulmonary disease (COPD), dementia, schizophrenia, long term use of antidepressants, anxiety, high cholesterol, hypertension, stroke and heart disease. The algorithms are described in Table 1. People with two or more of these 16 chronic conditions according to the diagnostic algorithms were identified as having multimorbidity. Socioeconomic status was measured by educational attainment based on the highest educational level of the individual, and grouped into four categories: primary and lower secondary school, vocational training, undergraduate education and postgraduate education [23].

**Table 1:** Algorithms used to define the 16 conditions

No.	Condition	Defined as a physical or mental health condition in the study	ICD-10 from the Danish National Patient Register	Definition
1	Diabetes	Physical		All persons included in the Danish Diabetes Register where the inclusion date is before the population date (1/1 2012) [22]
2	Cancer	Physical	C00-C43 or C45-C97	(DIAG) ^a^
3	Back pain	Physical	M40–54	(DIAG) ^a^
4	Osteoarthritis	Physical	M15-M19	(DIAG) ^a^
5	Osteoporosis	Physical	M80-M82and/or for persons aged 45 years and older contacts with the ICD-10 codes S22.0, S22.1, S32.0, S32.7, S32.8, S42.2, S42.3, S42.4, S42.7, S42.8, S42.9, S52.5, S52.6, S52.7, S52.8, S52.9, S62.0, S62.1, S72	(DIAG) ^a^ and/or (MEDICINE) ^b^ all medicine prescriptions with either ATC: M05B, G03XC01, H05AA02, H05AA03
6	Joint disease	Physical	M05, M06.0, M06.8, M07.0, M07.1, M07.3, M10.0, M10.9	(DIAG) ^a^
7	Allergies	Physical	J30 except J30.0	(DIAG) ^a^ and/or (MEDICINE) ^b^ all medicine prescriptions with either ATC: V01AA02, V01AA03; V01AA05, V01AA11; R01AC, R01AD, R06A, S01G, R01BA52.
8	Chronic obstructive pulmonary disease (COPD)	Physical	J40, J41, J42, J43, J44, J47, J96	(DIAG) ^a^ All patients of minimum 35 years of age at contact. And/or (MEDICINE1) ^b^ all medicine prescriptions with either ATC: R03AC, R03AK, R03BA, R03BB, R03CC, R03DA, R03DC, V03AN01. And/or (SERVICE10) ^c^ if patients have had a minimum of two lab services within the last 12 months. Lab services (80) 7113 (lung spirometer test), (80) 7121 (lung function test). MEDICINE and SERVICES are ignored if there are contacts with J45 or J46 diagnosis.
9	Dementia	Mental health	F00, G30, F01, F02.0, F03.9, G31.8B, G31.8E, G31.9, G31.0B	(DIAG) ^a^ All patients of minimum 60 years of age at contact and/or (MEDICINE) ^b^ all medicine with the prescriptions with the ATC N06D also for patients of minimum 60 years of age. NB: Only one prescription (in contrast to two in the other algorithms)
10	Schizophrenia	Mental health	F20, F21, F22, F25, F28, F29, F31	(DIAG) ^a^ and/or (MEDICINE) ^b^ all medicine prescriptions with either ATC: N05AX13, N05AX12, N05AH03, N05AX13, N05Ax08 NB: Only A-diagnosis not B-diagnosis
11	Long term use of antidepressants	Mental health		(MEDICINE) ^b^ At least three medicine prescriptions with the ATC N06A. NB: At least three prescriptions (instead of two) on deferent dates within the latest five year with at least 730 days (2 years) between the first and the last one. Patients with a schizophrenia diagnosis or with a dementia diagnosis are excluded from this algorithm.
12	Anxiety	Mental health	F40.1, F41.1	(DIAG) ^a^ and (MEDICINE) ^b^ At least three medicine prescriptions with the ATC N06A. NB: At least three prescriptions (instead of two) on deferent dates within the latest five year with at least 730 days (2 years) between the first and the last one. Patients with a depression diagnosis are excluded from this algorithm
13	High cholesterol	Physical	E78.0, E78.2, E78.4, E78.5	(DIAG) ^a^ and/or (MEDICINE) ^b^ all medicine prescriptions with the ATC C10
14	Hypertension	Physical	I10, I11, I12, I13, I15	(DIAG) ^a^ and (MEDICINE) ^b^ all medicine prescriptions with either ATC: C07B, C03A, C03B, C03E, C03X and/or (MEDICINE) ^b^ all medicine prescriptions with either ATC: C03C, C03D, C07A, C09 IF the person DOES NOT have hospital or outpatient contact with the ICD-10 codes I20.0, I21, I25.1, I50 and/or two medicine prescriptions with ATC: C08 IF the person DOES NOT have a hospital or outpatient contact with the ICD-10 codes I20-I25
15	Stroke	Physical	G45, G46, I60, I61, I62, I63, I64, I65, I66, I67, I68, I69	(DIAG) ^a^
16	Heart disease	Physical	I20, I21, I23, I24, I25, I50, I11, I13	(DIAG) ^a^ and/or (MEDICINE) ^b^ all medicine prescriptions with either ATC: C01A, C01B, C01D, C01E

^a^DIAG: All patients, any age unless specified otherwise, that have had a minimum of one hospital or outpatient contact, with one of the ICD-10 diagnosis codes of the condition within the last five years. Both ‘open’ (ongoing treatment) and ‘closed’ (finalized treatment) contacts, as well as primary (A), secondary (B) and additional (+) diagnosis are included

^b^MEDICINE: All patients that have had a minimum of two medicine prescriptions as defined by the ATC codes and/or indication codes within a period of 24 months, at last one time during the specific period from the time of interest. MEDICINE1 indicates that medication criteria have been included from 1 year from the time of interest and MEDICINE2 for 2 years

^c^SERVICE10: Patients that have had a minimum of one healthcare SERVICE during the last 10 years

### Statistical analysis

The analyses were based on Barnett et al.’s approach to presenting the prevalence of multimorbidity [4]. Prevalence was calculated and depicted graphically. In addition, odds ratios for multimorbidity were derived from logistic regression, where presence or absence of multimorbidity was regressed on gender, age, age squared and educational level. Odds ratios for multimorbidity for each variable were then adjusted for all other variables. Similarly, odds ratios for mental health conditions were derived from logistic regression, where presence or absence of mental conditions was regressed on gender, age, age squared, education and number of physical conditions. Odds ratios for mental health conditions for each variable were then adjusted for all other variables. Age squared was added as an independent variable after observing a nonlinear age effect. Standard deviations (SD) for demographic variables were derived using the binomial formula, and 95% confidence intervals were calculated. Standard deviations for odds ratios were extracted from logistic regression models and used to construct 95% confidence intervals. All analyses were carried out using R software version 3.2.2 [24].

## Results

The demographic characteristics of the Danish study population, the proportion of the population with multimorbidity and the proportion of the population with physical and mental health comorbidity are shown in Table 2. The study population included a slightly higher proportion of women (51.6%) than men (48.4%) and considerably more people with lower secondary education or vocational training (66.4%) than those with higher or postgraduate education (27.6%). Information about educational attainment was not available for 6.2% of the population. Of all individuals in the study population, 42.0% had one or more chronic conditions and 21.6% of the population had multimorbidity (two or more chronic conditions).

**Table 2:** Demographics and prevalence of multimorbidity and physical and mental health comorbidity

	% (n)	Mean number of morbidities (SD)	Percent (95% CI) with multimorbidity	Percent (95% CI) with physical and mental health comorbidity
All persons	100 (1,397,173)	0.85 (1.30)	21.6 (21.5–21.7)	4.9 (4.9–4.9)
Gender				
Female	51.6 (720,885)	0.93 (1.35)	23.7 (23.7–23.8)	6.3 (6.3–6.4)
Male	48.4 (676,288)	0.76 (1.24)	19.3 (19.2–19.4)	3.3 (3.3–3.4)
Age, years				
16–24	14.2 (198,024)	0.15 (0.38)	0.9 (0.9–0.9)	0.4 (0.4–0.4)
25–44	35.7 (498,313)	0.29 (0.61)	4.7 (4.7–4.8)	1.8 (1.8–1.8)
45–64	30.6 (428,043)	0.98 (1.25)	25.9 (25.8–26.0)	6.0 (5.9–6.0)
65–84	17.1 (238,864)	2.09 (1.65)	58.6 (58.5–58.7)	10.4 (10.4–10.5)
> 84	2.4 (33,929)	2.70 (1.64)	75.1 (75.0–75.2)	23.6 (23.6–23.7)
Educational attainment				
Lower secondary	25.0 (348,828)	1.04 (1.47)	26.9 (26.8–26.9)	6.7 (6.6–6.7)
Vocational	41.4 (579,100)	0.88 (1.30)	22.5 (22.5–22.6)	4.7 (4.6–4.7)
Higher education	16.0 (223,643)	0.74 (1.16)	18.6 (18.5–18.6)	4.3 (4.2–4.3)
Postgraduate education	11.7 (163,787)	0.57 (1.00)	13.5 (13.5–13.6)	2.7 (2.7–2.8)
Number of chronic conditions				
0	58.0 (809,920)	−	−	−
1	20.4 (285,496)	−	−	−
2	9.9 (137,751)	−	−	17.4 (17.2–17.6)
3	6.1 (85,680)	−	−	20.2 (19.9–20.4)
4	3.3 (45,442)	−	−	27.7 (27.3–28.1)
5	1.5 (20,785)	−	−	38.2 (37.6–38.9)
6	0.6 (8186)	−	−	48.6 (47.5–49.7)
7	0.2 (2819)	−	−	60.3 (58.5–62.1)
8+	0.1 (1094)	−	−	70.7 (68.1–73.4)

**Fig. 1:**
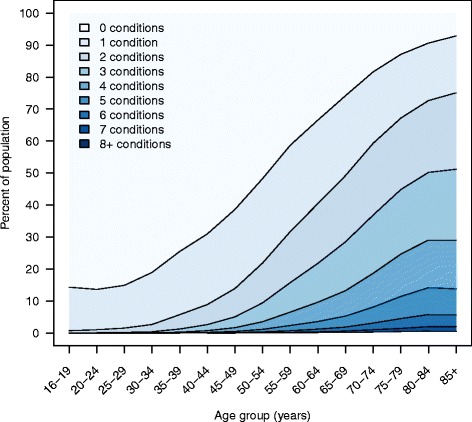
Number of chronic conditions by age group

**Fig. 2:**
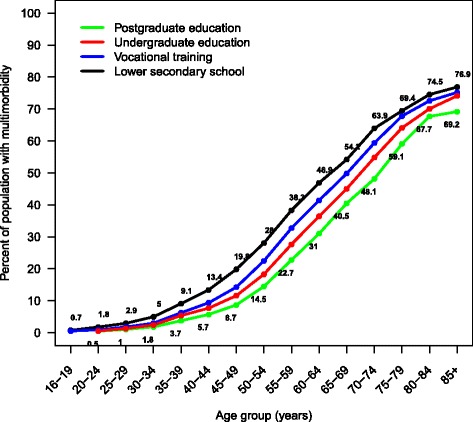
Prevalence of multimorbidity by age and educational attainment

As shown in Fig. 1, the number of chronic conditions per person and the proportion of people with multimorbidity increased substantially with age. Half of the population aged 65 and above had multimorbidity. The crude prevalence of multimorbidity was negatively associated with educational attainment: 26.9% (95% confidence interval [CI], 26.8–26.9) among those with lower secondary education versus 13.5% (95% CI, 13.5–13.6) among people with postgraduate education (Table 2). People aged 65–69 years with postgraduate education had rates of multimorbidity that were similar to those who were 10 years younger with the lowest level of educational attainment (40.5 vs. 38.3, Fig. 2). Odds ratios adjusted for gender, age and age squared showed that the prevalence of multimorbidity was negatively associated with educational attainment. People with higher educational levels had lower adjusted ORs for multimorbidity (vocational training OR 0.79; 95% CI, 0.78–0.79; undergraduate education OR 0.63; 95% CI, 0.61–0.64; postgraduate education OR 0.51; 95%, 0.49–0.50), compared to people with lower secondary education.

**Fig. 3:**
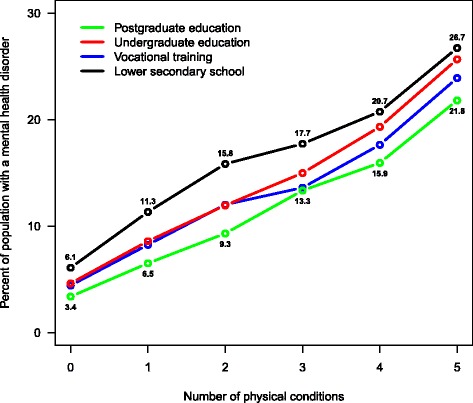
Prevalence of physical and mental health comorbidity and the association with educational attainment

Of all population members, 4.9% (95% CI, 4.9–4.9) had both a physical and a mental health condition. This was the case for 22.6% of people with multimorbidity. The prevalence of physical and mental health comorbidity was higher in women (6.3%; 95% CI, 6.3–6.4) than in men (3.3%; 95% CI, 3.3–3.4) and approximately 50 times higher among older persons than younger ones (Table 3). Even though older people were much more likely to have physical and mental health comorbidity, the absolute number of individuals with physical and mental health comorbidity was greater among younger people (35,323 persons aged <65 years vs. 32,919 persons ≥65 years). As shown in Table 2, the prevalence of multimorbidity and physical and mental health comorbidity among people with lower secondary education was more than twice that of those with postgraduate education. The presence of a mental health condition was strongly associated with the number of physical conditions; for example, individuals with five or more physical conditions had an adjusted OR for a mental health condition of 3.9 (95% CI, 3.8–4.1), compared to those with no physical conditions (Table 2). Figure 3 shows the association between educational attainment and the presence of any mental health condition by the number of physical conditions. For individuals with zero to 2 physical conditions, the four educational groups demonstrated three levels of prevalence, with relative differences being independent of the number of physical conditions, vocational training and undergraduate education having similar prevalence and prevalence decreasing with the level of education. For individuals with four or more physical conditions, prevalence was different for each of the four educational groups, with the absolute differences independent of the number of physical conditions and a decrease with level of education similar to that among individuals with zero to 2 physical conditions. Thus, Fig. 3 shows that, independent of the number of physical conditions, the risk of having a mental health condition is higher for people with low educational attainment compared to those with high educational attainment.

**Table 3:** Odds ratios and 95% CIs for any mental health condition by age, gender, socioeconomic status and number of physical conditions

	Odds Ratio (95% CI)	Adjusted Odds Ratio (95% CI)^a^
Male (vs. female)	0.58 (0.58–0.59)	0.63 (0.62–0.64)
Age^b^	1.34 (1.34–1.35)	1.47 (1.44–1.49)
Age squared	1.03 (1.03–1.03)	0.98 (0.98–0.98)
Educational attainment		
Postgraduate education	Reference	Reference
Undergraduate education	1.45 (1.41–1.49)	1.24 (1.20–1.27)
Vocational training	1.48 (1.44–1.51)	1.26 (1.23–1.30)
Lower secondary school	2.13 (2.08–2.18)	1.75 (1.70–1.79)
Number of physical conditions		
0	Reference	Reference
1	2.07 (2.04–2.11)	1.61 (1.58–1.63)
2	3.19 (3.13–3.25)	2.04 (1.99–2.08)
3	3.86 (3.77–3.94)	2.32 (2.26–2.37)
4	4.98 (4.85–5.11)	2.87 (2.79–2.96)
≥ 5	7.12 (6.98–7.31)	3.93 (3.80–4.07)

^a^Adjusted for all other model variables

^b^ORs are per 10-year increase in age

Comorbidities of people diagnosed with heart disease, diabetes, chronic obstructive pulmonary disease or cancer were common for people with low educational attainment (Table 4). People with the lowest educational level were more likely to have heart disease, diabetes, COPD and depression (measured as long term use of antidepressant medications) as comorbidities compared to people with the highest educational attainment (Table 4).

**Table 4:** Selected comorbidities in people with four common disorders by educational attainment

Persons with this condition	Persons who also have this condition (%)					
	Heart disease	Diabetes	COPD	Stroke	Use of antidepressant medications	Dementia
Heart disease						
Postgraduate	-	17,3	15,9	7,4	8,5	3,0
Lower secondary	-	30,0	27,9	10,9	12,8	4,9
Diabetes						
Postgraduate	13,9	-	11,8	4,3	8,2	1,5
Lower secondary	21,6	-	19,6	6,8	11,8	2,8
COPD						
Postgraduate	8,0	7,3	-	2,5	8,5	1,0
Lower secondary	18,2	18,2	-	6,5	15,0	2,7
Cancer						
Postgraduate	8,1	7,1	10,4	3,0	6,7	1,0
Lower secondary	16,2	16,2	20,3	5,9	11,4	2,6

## Discussion

Our results reveal that multimorbidity is common in the Danish population and is substantial among the elderly; more than half of the population aged 65 years and up has at least two chronic conditions and one-fourth of the population between the ages of 45 and 64 years is living with multimorbidity. Furthermore, multimorbidity is more prevalent among women than among men and twice as prevalent in the population with the lowest educational attainment, compared to those with postgraduate education. Accordingly, the results show that multimorbidity is negatively associated with educational attainment. The risk of having a mental health condition was higher for women and for people with lower secondary education or vocational training and increased with age and the number of physical conditions. Further, the results showed that comorbidities of heart disease, diabetes, chronic obstructive pulmonary disease or cancer were common for people with low educational attainment.

Our results are comparable to those of other studies finding that age, lower socioeconomic status and gender are associated with multimorbidity [4, 18, 25–27], and the multimorbidity prevalence we report is comparable to that reported by Orueta et al. [25] and Barnett et al. [4]. Orueta et al. found that 23.6% of the total Basque population had two or more chronic conditions [25], and Barnet et al. found that 23.2% of all patients in Scotland had two or more chronic conditions [4], compared to 21.6% in our study. Both studies found the same prevalence patterns across socioeconomic groups [4, 25]. Orueta et al. used health administrative databases from both primary care and hospitals and a list of 52 chronic conditions [25] and Barnet et al. used data from primary care to identify chronic conditions and included 40 conditions [4]; in contrast, we used data from both the primary and secondary sectors and included only 16 diagnoses. The study populations in Orueta et al.’s and Barnett et al.’s studies included children aged 0–15 years. This group was not included in our study population because very few children under the age of 16 years have multimorbidity [4]. Further, both studies defined socioeconomic status by the area in which a person lived [4, 25], whereas individual educational attainment was used as an indicator of socioeconomic status in this study. We believe that similar patterns identified in several European populations reflect the fact that the number of included conditions does not affect the overall results because the most prevalent conditions are included in all studies. Similarly, including diagnoses from both primary and secondary sectors, compared to including only diagnoses from the primary care sector, does not substantially affect the results as long as the primary care sector diagnoses conditions. This is consistent with Harrison et al., who concluded that multimorbidity defined as two or more diseases can be accurately measured using varying definitions that include as few as 12 prevalent chronic conditions [7]. Thus, we believe that the comparable patterns of multimorbidity prevalence suggest that similar patterns are likely to be found in other Western European countries.

In keeping with this, our findings related to the relationship of multimorbidity to age are also consistent with recent studies from the Netherlands [10] and Ireland [28], both based on data from general practice, measuring multimorbidity as two or more co-occurring chronic conditions and using a list of 29 and 147 chronic conditions, respectively. Our results are also comparable with those of an English study with regard to prevalence and age and socioeconomic status [29]. The study is based on data from primary care and uses two different approaches to define multimorbidity. The prevalence of physical and mental health comorbidity of 4.9% in our study was lower than the 8.3% reported by Barnett et al. in a Scottish population [4] and the 7.9% reported by Bobo et al. in a US population [17]. Chronic conditions in our study were identified by algorithms based on ICD-10 codes from the hospital system, medication prescriptions and some services provided by primary care; consequently, mental health conditions that were not treated with medications were not identified. This may explain the differences in reported prevalence of physical and mental health comorbidity because both studies noted above [4, 17] included data from primary care, where we assume that diagnoses of mental health conditions are not contingent on medication use.

The results also revealed that the risk of having a mental health condition increases with age, number of physical health conditions and educational attainment which is consistent with Barnett et al.’s results [4]. In the same population, McLean et al. [30] also found a strong association between prevalence of multimorbidity and socioeconomic deprivation. Several studies have investigated the association between childhood conditions and the development of chronic conditions [31–33] and conclude that early life conditions have a lasting influence on adult health. Tomasdottir et al. state that allostatic overload can be the underlying mechanism behind this association, providing a route by which childhood adversities become biologically embodied [33]. Thus, in our study, low educational attainment can be an indicator of poor childhood conditions. In addition, factors such as different working environments and differences in health behaviors such as smoking and diet can contribute to the educational differences in the prevalence of multimorbidity [34]. In keeping with Barnett et al. [4], we found that comorbidities for heart disease, diabetes, chronic obstructive pulmonary disease or cancer were more common in people with low educational attainment [4]. In contrast, Barnett et al. could not demonstrate socioeconomic effect for stroke and dementia. A likely explanation for the differences between these results is that our study used individual educational attainment as an indicator of socioeconomic status, whereas Barnett et al. used socioeconomic deprivation of the area in which a patient lived to define socioeconomic status [4].

Strengths of our study include a relatively large population consisting of all adults from the Danish Capital Region and including data from both the primary and secondary sectors. Study limitations include the use of algorithms to identify chronic conditions. Previous studies indicate that the algorithms do not capture all persons with rheumatoid arthritis, osteoarthritis, back conditions, lung diseases, mental health disorders and allergies [11]; hence, the prevalence of multimorbidity may be underestimated here. In addition, the use of algorithms based on register data from the healthcare system means that the prevalence of the included chronic conditions might be underestimated because the register includes only people who are in contact with the healthcare system. Not surprisingly, the prevalence of multimorbidity found in this study is lower than studies from European countries using information from self-reported questionnaires to identify chronic conditions [9, 35, 36].

## Conclusions

Our results emphasize the magnitude of the prevalence of multimorbidity and reveal that prevalence patterns of multimorbidity found in this study are comparable to those of other European populations, which may indicate that the patterns are also likely to be found in other Western European countries. Furthermore, the results support evidence showing that variations in the number of chronic conditions used to calculate the prevalence of multimorbidity are immaterial.

Persons living with multimorbidity in the Danish Healthcare System often receive care for their different conditions from different health care providers [37]. Often, mental health conditions are not taken into consideration when providing care for physical health conditions [37]. The high proportion of the population that has physical and mental health comorbidity highlights the need to ensure that healthcare systems deliver care that takes both physical and mental health into account. Furthermore, the higher prevalence of multimorbidity among persons with low educational attainment emphasizes the importance of a healthcare system providing care that is beneficial to all, regardless of socioeconomic status and educational attainment. Finally, the findings highlight the need for additional research into life-course socioeconomic pathways associated with the absence of morbidity and the occurrence of multimorbidity later in life.
